# Evaluation of Selected Parameters of the Specific Immune Response against *Pseudomonas aeruginosa* Strains

**DOI:** 10.3390/cells11010003

**Published:** 2021-12-21

**Authors:** Michał Dzik, David Aebisher, Alina Olender, Jacek Tabarkiewicz

**Affiliations:** 1 Department of Clinical Immunology, Faculty of Medicine, Medical University of Lublin, 20-095 Lublin, Poland; michaldzik@wp.pl; 2 Institut Asclépiade, 10410 Saint-Parres-aux-Tertres, France; 3 Centre for Innovative Research in Medical and Natural Sciences, Medical College of Rzeszów University, University of Rzeszów, 35-959 Rzeszow, Poland; daebisher@ur.edu.pl; 4 Chair and Department of Medical Microbiology, Faculty of Medicine, Medical University of Lublin, 20-095 Lublin, Poland; alina.olender@umlub.pl; 5 Department of Human Immunology, Institute of Medical Sciences, Medical College of Rzeszów University, University of Rzeszów, 35-959 Rzeszow, Poland

**Keywords:** *Pseudomonas aeruginosa*, dendritic cells, HLA-DR, Th17 cells

## Abstract

The immune response to *Pseudomonas aeruginosa* strains could be influenced by differences in antibiotic resistance and virulence. At the present time, it is unclear which type of immune responses enables uncontrolled invasion of opportunistic pathogens. The conditional pathogenicity of *Pseudomonas aeruginosa* served as an inspiration to begin a study on this bacterium. The aim of this study was to gain insight into selected parameters describing immune responses with regards to the adaptable agents of this pathogen. For the analysis of the specific immune response, the potential of *Pseudomonas aeruginosa* to stimulate lymphocytes, including Th17 lymphocytes, dendritic cells and other components of the adaptive immune response, was examined. The highest percentage of CD83+CD1a-HLA-DR++ cells was found after stimulation with lysates of strains isolated from the patients with severe systemic infection. We found statistically significant differences in percentages of HLA-DR+ PBMCs and MFI of HLA-DR between groups of *Pseudomonas aeruginosa* strains isolated from the patients with different clinical courses of infection. Our results suggest that the clinical course and outcomes of *Pseudomonas aeruginosa* infections are not associated with impairment of the specific immune response.

## 1. Introduction

The human immune system is constantly interacting with the external environment, as well as many pathogens. The nature of this interaction is dynamic—it changes over time; it also intensifies and specifies both the way in which the attacked organism fights and eliminates the pathogen, and, at the same time, pathogen virulence changes. Moreover, pathogenic bacteria are not only able to effectively oppose immune mechanisms, but, at the same time, are able to resist additional external factors, such as the antibiotics used. One important bacterium, which shows special adaptive properties, is Gram-negative *Pseudomonas aeruginosa*. *Pseudomonas aeruginosa* is commonly recognized as an opportunistic pathogen and is not pathogenic in healthy populations [1]. Occasionally, exceptions are described in the literature in cases of inhalation exposure to very high levels of bacteria that are not relevant to day-to-day clinical practice [2]. On the other hand, it is a pathogen of great clinical importance because it causes opportunistic infections and thus infects immunocompromised individuals. *Pseudomonas aeruginosa* is also a very important epidemiological problem of nosocomial infections characterized by a treatment-resistant course, including pneumonia, and including those associated with the use of artificial ventilation, intra-abdominal infections after surgery, burn wounds, meningeal infections and sepsis. This bacterium is the main epidemiological factor of chronic pneumonia in people with cystic fibrosis (CF), and the long-term immune response of these patients to the presence of *Pseudomonas aeruginosa* causes the final destruction of the lungs by inflammatory factors, tragically closing the pathogen–host vicious cycle [3]. Another important ophthalmological problem is keratitis, which quickly becomes aggressive and erosive [4,5,6,7,8]. One of the characteristic features of *Pseudomonas aeruginosa* is its high virulence-modifying and immune-bypassing ability, so it is interesting to gain additional insight into how *Pseudomonas aeruginosa* affects host defense mechanisms. The conditional nature of *Pseudomonas aeruginosa* pathogenicity was the inspiration to begin researching this bacterium. It is not clear at this time which changes in the immune system allow opportunistic pathogens to proliferate and initiate a clinically apparent invasion. This study attempts to understand differences which could be associated with the invasive course of *Pseudomonas aeruginosa* infections and to answer the question as to what extent selected elements of specific immune responses are influenced by different adaptation factors of this pathogen, e.g., antibiotic resistance, type of colony growth or genetic regulation of virulence. We chose to analyze the reactivity of well-functioning immune system components against *Pseudomonas aeruginosa* strains isolated from different anatomical locations that differ in antibiotic resistance and virulence phenotype. For this analysis, the potential of *Pseudomonas aeruginosa* for stimulation of dendritic cell maturation, HLA-DR expression, Th17 lymphocytes differentiation and release of pro- and anti-inflammatory cytokines was evaluated.

## 2. Materials and Methods

### 2.1. Characteristics of the Patients Studied

The study included 15 patients (7 women and 8 men) from whom *Pseudomonas aeruginosa* was isolated. The average age of the respondents was 60.8 ± 1.41 years. Ten patients were hospitalized in the Independent Public Clinical Hospital No. 4. in Lublin and the pathogen was isolated in the hospital lab. Based on the course of the disease, which was determined by an analysis of medical documentation obtained from the archive of SPSK4, five patients were selected in two groups:

Group A—5 patients with severe systemic infection with *Pseudomonas aeruginosa*, which eventually resulted in patient death.

Group B—5 patients with a urinary tract infection caused by *Pseudomonas aeruginosa*, with successful completion (cure or remission of recurrent infections).

Five were outpatients, and isolation of *Pseudomonas aeruginosa* took place in the microbiological laboratory of Luxmed in Lublin and were included as group C—5 outpatients with asymptomatic colonization of upper respiratory tract by *Pseudomonas aeruginosa*.

The biological samples taken from patients were as follows: Group A—peripheral blood, which was collected into media of the BacT/Alert system (bioMérieux, Marcy l’Etoile, France); Group B—urine; Group C—nose and throat swabs. The cultures of the materials were prepared in accordance with routine microbiological diagnostics. *Pseudomonas aeruginosa* strains were grown in Columbia Agar supplemented with 5% Sheep Blood (bioMérieux, Marcy l’Etoile, France) and MacConkey Agar (bioMérieux, Marcy l’Etoile, France) as a homogeneous, abundant monoculture of blood culture and monoculture urine growth above 105 CFU/mL (significant bacteriuria). From nose and throat swabs, *Pseudomonas aeruginosa* was cultured from the throat in an average of 5–10 colonies, which indicated colonization against the dominant growth of the physiological flora. The identification of the species *Pseudomonas aeruginosa* was performed by using commercial APINE identification tests (bioMérieux, Marcy l’Etoile, France). The purity of the test strains was checked under a microscope (Gram-stained slide) and by analyzing the morphology of bacterial colonies grown on Columbia agar supplemented with 5% sheep blood (bioMérieux, France) and MacConkey agar (bioMérieux, France).

Blood samples of healthy volunteers with a mean age of 29.9 ± 7.5 years (3 women and 7 men) were used to evaluate selected parameters of the immune response to the above strains of *Pseudomonas aeruginosa*. The healthy volunteers did not suffer from any bacterial or viral infection for at least one month prior to the date of blood collection and did not receive any medication that may affect the immune system. Volunteers also did not suffer from chronic diseases or allergies.

The study was conducted in accordance with a protocol approved by the Bioethics Committee of the Medical University of Lublin in resolution KE-0254/184/2013. Informed consent was obtained from all subjects involved in the study.

### 2.2. Preparation of Test Material

Bacteria obtained from diagnostic laboratories were frozen at −80 °C, using the Microbank system (Pro-Lab Diagnostics, Round Rock, TX, USA), which enables safe freezing and reclamation of frozen cells. As the study progressed, live suspensions and their lysates were prepared from the frozen strains of *Pseudomonas aeruginosa*.

### 2.3. Preparation of Suspensions of Living Bacteria

Pure bacterial cultures were isolated on solid media plates—Columbia Agar (bioMerieux, France) was prepared according to the following recipe: 400 mL agar solution with manufacturer’s recommended concentration was sterilized by autoclaving, and 20 mL of sheep blood (Biomed Lublin, Lublin, Poland) was added. After 24 h of culture, the bacteria were again sieved to the same medium, and after 24 h, pure culture suspensions were prepared in Dulbecco’s PBS with Ca^2+^ and Mg^2+^ (PAA, Pasching, Austria). A density of 1 McFarland (McF, about 3 × 10^8^ CFU/mL) was measured by using a Densimat photometer (BioMerieux, Marcy l’Etoile, France) to determine the concentration of the solution by measuring the intensity of light scattering. 

Based on the type of growth of *Pseudomonas aeruginosa* colonies on solid media, the mucoid and non-mucoid types were differentiated. Mucus type growth was observed in strains 2, 3, 5, 7, 9, 11 and 14.

### 2.4. Preparation of Bacterial Lysates

Live bacterial suspensions prepared as above were rapidly frozen at −80 °C and thawed to pre-disintegrate the bacterial cells. They were subsequently sonicated in a Branson Ultrasonics 2510 (Branson Ultrasonics, Brookfield, CT, USA) sonicator at a temperature of 46 °C for 30 min. The resulting lysates were filtered through 0.22 μm Millex GP filters (Merck Millipore, Burlington, MA, USA), separated into Eppendorf tubes of 1.5 mL and frozen at −80 °C. The whole procedure of lysates ensured the sterility of the final product.

### 2.5. Determination of Protein Concentration in Bacterial Lysates

The total protein concentration in the lysates was determined with the Bradford protein assay, using the Bradford Reagent (Sigma-Aldrich, St. Louis, MO, USA) and the SmartSpec Plus spectrophotometer (Bio-Rad, Hercules, CA, USA) by measuring the absorbance of the samples at 595 nm. The exact protein concentration of the samples was determined by interpolation from a standard curve made by measuring the absorbance of a dilution series of bovine serum albumin (BSA) (Sigma-Aldrich, USA) of known concentrations within the linear response range of the Bradford protein assay.

### 2.6. Pseudomonas aeruginosa Virulence Genes Determination

Using the polymerase chain reaction (PCR) method, the presence of the *algD, lasB, toxA, plcH, plcN, exoS* and *nan1* genes was determined in the examined *Pseudomonas aeruginosa* strains. The primers sequences (Genomed, Warszawa, Poland) are summarized in Supplementary Materials Table S1.

Then 1 kb markers (New England BioLabs, Ipswich, MA, USA) were used for *algD* and *nan1* genes and 100 bp for other genes. The following program was used on the iCycler Thermal Cycler (BIO-RAD, Hercules, CA, USA): initial denaturation—94 °C for 3 min, 30 cycles: 94 °C after 30 s (denaturation), 55 °C for 1 min. (primer annealing), 72 °C for 1 min. Then 30 s (elongation) and one stage of final extension: 72 °C for 5 min.

Each gene was amplified separately. The previously developed procedure for detecting the presence of these genes, as described in the literature, was followed [9].

On the basis of the presence of individual genes, 4 groups were defined in the tested material (Table 1). The groups defined by the presence of virulence genes did not coincide with the groups defined by the anatomical location of the infection.

### 2.7. Isolation of Peripheral Blood Mononuclear Cells (PBMC)

Peripheral blood was diluted 1:2 with PBS w/o Ca^+2^ or Mg^+2^ (PAA, Austria) and then overlaid on Gradisol L (Aqua Medica, Łódź, Poland) and centrifuged for 20 min, with an acceleration of 700× *g*. The collected PBMCs were washed twice in the same PBS for 7 min at 700× *g*. Then the collected cells were suspended in 3 mL of AIM V Serum Medium (Life Technologies Corporation, Carlsbad, CA, USA) and the cells were counted and their viability determined by using Trypan Blue (Trypan Blue stain 0.4%, Invitrogen, Molecular Probes, Eugene, OR, USA) and a countess cell counter (Invitrogen, Molecular Probes, USA). It was assumed that cell viability could not be less than 90%.

### 2.8. Establishing a Culture of Isolated PBMC

Cells were cultured in Cellstar six-well plates (Greiner Bio-One, Frickenhausen, Germany) by adding 2 × 10^6^ cells to each well and 3 mL of AIM V Serum Free Medium (Life Technologies Corporation, Carlsbad, CA, USA), and then adding one of 15 lysates (in a volume corresponding to 30 µg of total protein per 1 mL of culture) to each well. The dose of bacterial lysates was set after optimization of protocol. The cells were cultured in an incubator at 5% CO_2_, 37 °C and 95% humidity for 72 h.

Then 0.5 mL of the suspension was removed from each well for the determination of dendritic cells, and to the remainder of the culture the same volume of lysates was added again to restore the lymphocytes and Brefeldin A (Sigma-Aldrich, St. Louis, MO, USA) was added in the amount recommended by the manufacturer. After carefully mixing the contents, the cells were incubated for another 6 h. The addition of Brefeldin A was to block intracellular transport within the Golgi reticulum and to inhibit secretion of IL-17A into the medium by lymphocytes, as the latter procedures are based on the intracellular determination of IL-17A.

After restimulation, the cultured cells were separated from the supernatants by centrifugation for 7 min at 700× *g*. The collected supernatants were frozen at −80 °C for determination of the pro- and anti-inflammatory cytokine profile by cytometric methods.

### 2.9. Analysis of Dendritic Cell Phenotype after Stimulation with Bacterial Lysates

After 72 h of PBMC stimulation with bacterial lysates, maturation of dendritic cells was evaluated with the use of flow cytometry. We excluded from analysis cultures with viability lower than 90%. Unstimulated PBMC were used as negative control, and ionomycin (Sigma-Aldrich, St. Louis, MO, USA) stimulated cells were used as positive control. Cells were stained with FITC-labeled Mouse IgG1 κ anti-human CD83 (Clone HB15e, BD Biosciences, San Jose, CA, USA), PE Mouse IgG1 κ anti-Human CD1a (Clone HI149, BD Biosciences, San Jose, CA, USA) and PE-Cy5 Mouse IgG2a κ anti-Human HLA-DR (Clone G46-6, BD Biosciences, San Jose, CA, USA). Acquisition and analysis were carried out in the Guava EasyCyte (Millipore, USA) cytometer with InCyte software. CD83+CD1a-HLA-DR++ cells were counted as mature DCs, CD83+CD1a+HLA-DR+ as partially matured and CD83-CD1a+HLA-DR+/low as immature. We also evaluated percentages of HLA-DR+ PBMC and expression of HLA-DR on PBMC shown as mean fluorescence intensity (MFI). The representative case of gating strategy and analysis is shown in the Supplementary Materials Figure S1. 

### 2.10. Analysis of Percentages of Th17 Lymphocytes (CD3+CD4+IL-17+) Stimulation with Bacterial Lysates

Cells from culture, after restimulation with bacterial lysates and incubation with Brefeldin A, were washed and incubated with PE-Cy5 Mouse IgG2a κ Anti-Human CD3 (Clone HIT3a, BD Biosciences, San Jose, CA, USA) and FITC Mouse BALB/c IgG1 anti-human CD4 (Clone L120, BD Biosciences, San Jose, CA, USA) in the dark at room temperature for 20 min. After incubation, 100 μL IC Fixation Buffer (eBioscience, San Diego, CA, USA) was added and incubated for another 20 min, and then 2 mL permeabilization buffer (eBioscience, San Diego, CA, USA) was added and centrifuged (7 min, 700× *g*). Rinse operation with permeabilization buffer was repeated. In the next step, 100 μL permeabilization buffer and 10 μL Mouse IgG1κ anti-human-IL-17A-PE (Clone CZ8-23G1, Miltenyi Biotec, Bergisch Gladbach, Germany ) were added to the tubes and incubated for 20 min in the dark at room temperature. After this time, it was rinsed with permeabilization buffer and centrifuged (7 min, 700× *g*). The resulting cells were suspended in 1 mL Flow Cytometry Staining Buffer (eBioscience, San Diego, CA, USA). Acquisition and analysis were carried out in the Guava EasyCyte (Millipore, Burlington, MA, USA) cytometer and InCyte software. Unstimulated and incubated with Brefeldin A cells were used as negative control; PBMC stimulated with ionomycin and incubated with Brefeldin A served as positive control. We excluded from analysis cultures with viability lower than 90%. The representative case of gating strategy and analysis is shown in the Supplementary Materials Figure S2.

### 2.11. Analysis of Pro and Anti-Inflammatory Cytokines Levels in PBMC Culture Supernatants Stimulated with Bacterial Lysates

Determination of pro- and anti-inflammatory cytokines levels in bacterial lysate stimulated PBMC culture supernatants was performed by the cytometric method of CBA (Cytometric Bead Array), using a commercial BD CBA Human Inflammatory Cytokines Kit (BD Biosciences, San Jose, CA, USA) according to the procedure specified by the manufacturer. The study was performed on a BD FACSCalibur dual-laser flow cytometer (Becton Dickinson, San Jose, CA, USA), using CellQuest Pro data acquisition and analysis software (Becton Dickinson, San Jose, CA, USA). We analyzed concentrations of IL-1β, IL-6, IL-8, IL-10 and TNF. Measurements of IL-12 level determinations were excluded from further analysis, as the obtained results were below the detection level in all tested samples.

### 2.12. Statistical Analysis

Statistical analysis of the results was carried out by using the Statistica 10 PL software (StatSoft Polska Sp. z o.o., Kraków, Poland). Results are presented as median, interquartile range (IQR) and minimal and maximal values. Because the variables had no normal distribution, as evidenced by the Shapiro–Wilk test, non-parametric tests were used for analysis. The Mann–Whitney U test was used for comparing the differences between 2 groups and the Friedman’s ANOVA with Tukey’s post hoc tests for comparison between 3 and more groups. Results were considered statistically significant at *p* ≤ 0.05.

## 3. Results

### 3.1. Evaluation of Induction of Dendritic Cell Maturation

**Figure 1:**
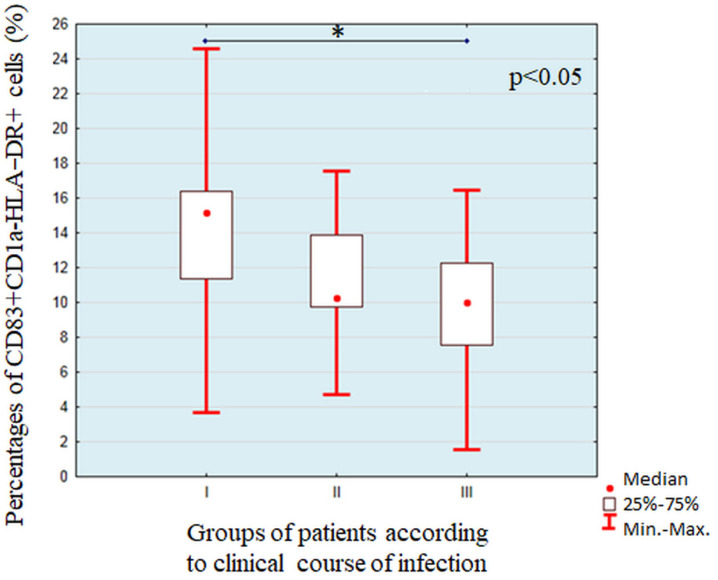
Comparison of percentages of fully matured dendritic cells after stimulation with *Pseudomonas aeruginosa* strains isolated from patients with different course of disease. The line show statistically significant differences shown with the use of *post-hoc* tests, * symbolizes *p* < 0.05.

By use of flow cytometry, we evaluated the influence of individual bacterial lysates on dendritic cell maturation. The results obtained from individual patients are summarized in Supplementary Materials Tables S2–S4. We did not find any statistically significant differences in percentages of dendritic cells on any particular stage of maturation between *Pseudomonas aeruginosa* strains forming mucoid or non-mucoid colonies. Neither the differences in antibiotic susceptibility nor profile of genes associated with virulence influenced maturation of DCs. We found statistically significant (χ^2^ = 6.88, *p* = 0.0319) differences in percentages of fully matured DCs after stimulation with *Pseudomonas aeruginosa* strains isolated from patients with different courses of disease (Figure 1). The highest percentage of CD83+CD1a-HLA-DR++ cells was found after stimulation with lysates of strains isolated from the patients with severe systemic infection with *Pseudomonas aeruginosa*, which resulted in patient death, and post hoc analysis showed a significant difference between strains isolated form patients with fatal outcome and patients with asymptomatic colonization of upper respiratory tract. We did not find any significant differences between percentages of partially matured and immature dendritic cells.

### 3.2. Analysis of HLA-DR Expression on PBMC

**Figure 2:**
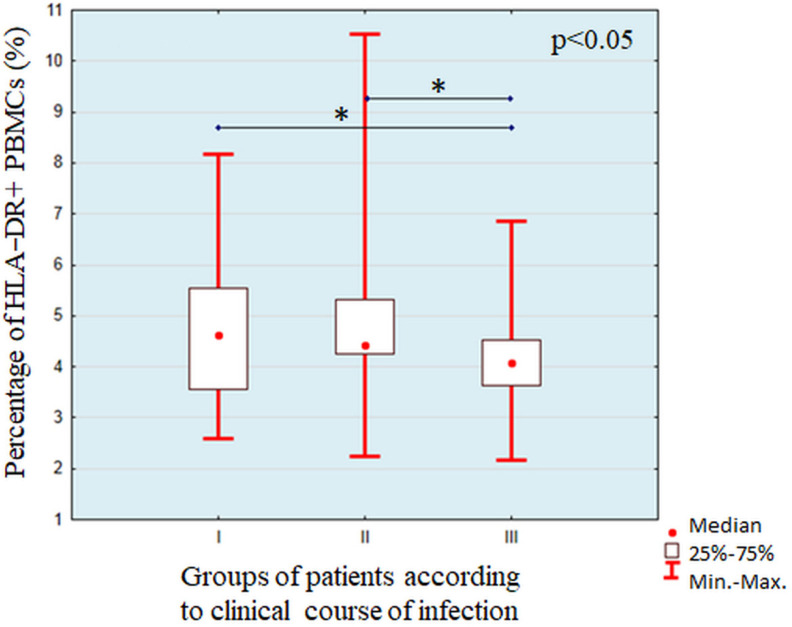
Analysis of percentages of HLA-DR+ PBMC after stimulation with *Pseudomonas aeruginosa* strains isolated from the patients with different clinical course of infection. The lines show statistically significant differences shown with the use of post-hoc tests, * symbolizes *p* < 0.05.

**Figure 3:**
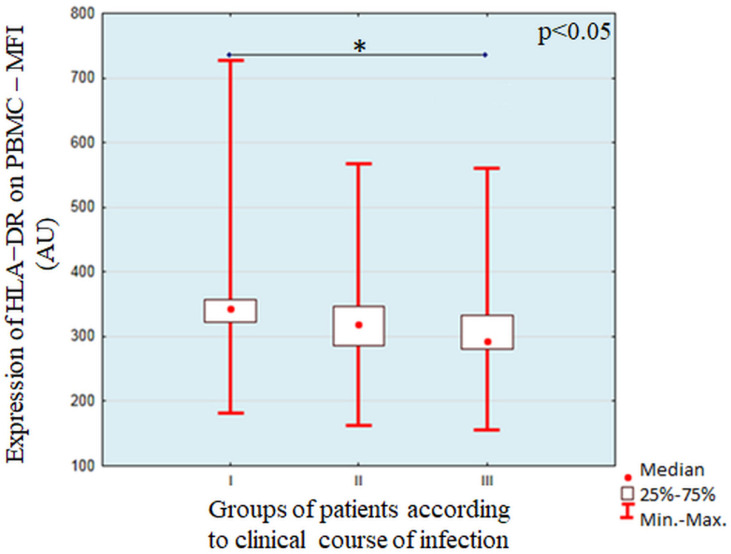
Analysis of HLA-DR expression on PBMC – MFI after stimulation with *Pseudomonas aeruginosa* strains isolated from patients with different clinical course of infection. The line show statistically significant differences shown with the use of *post-hoc* tests, * symbolizes *p*<0.05.

The individual characteristics of induction of HLA-DR expression on PBMC after stimulation with lysates of individual strains of *Pseudomonas aeruginosa* are summarized in Supplementary Materials Tables S5 and S6. We did not find any statistically significant associations between expression of HLA-DR on PBMC expressed as percentage of HLA-DR+ cells in PBMC gate or as MFI (mean fluorescence intensity) with antibiotic sensitivity of examined strains, mucoid or non-mucoid phenotypes, as well as profile of genes associated with virulence. We found significant differences in the percentage of HLA-DR+PBMCs (χ^2^ = 10.88, *p* = 0.0043) and MFI of HLA-DR (χ^2^ = 9.55, *p* = 0.0084) between groups of *Pseudomonas aeruginosa* strains isolated from the patients with different clinical course of infections. A detailed paired analysis is shown in Figure 2 and Figure 3.

### 3.3. Analysis of Percentages of CD3+CD4+ Cells

The comparisons of percentages of CD3+CD4+ cells after stimulation with lysates of individual strains of *Pseudomonas aeruginosa* are summarized in Supplementary Materials Table S7. We did not find any statistically significant associations between percentages of CD3+CD4+ cells with antibiotic sensitivity of examined strains, mucoid or non-mucoid phenotypes of colonies, profile of genes associated with virulence or clinical course of infection.

### 3.4. Analysis of Percentages of Th17 Cells

The comparisons of percentages of CD3+CD4+IL-17+ cells after stimulation with lysates of individual strains of *Pseudomonas aeruginosa* are summarized in Supplementary Materials Table S8. We did not find any statistically significant associations between percentages of CD3+CD4+IL-17+ cells with antibiotic sensitivity of examined strains, mucoid or non-mucoid phenotypes of colonies, profile of genes associated with virulence or clinical course of infection.

### 3.5. Analysis of Pro- and Anti-Inflammatory Cytokines Levels in PBMC Culture Supernatants Stimulated with Bacterial Lysates

A comparison of concentrations of IL-1β, IL-6, IL-8, IL-10 and TNF after stimulation with the lysates of individual strains of *Pseudomonas aeruginosa* did not reveal any statistically significant differences. We did not find any statistically significant associations between concentrations of IL-1β, IL-6, IL-8, IL-10 and TNF with antibiotic sensitivity of examined strains, mucoid or non-mucoid phenotypes of colonies, profile of genes associated with virulence or clinical course of infection.

## 4. Discussion

In this work, an analysis was conducted to elucidate the question of which factor is more important, the immune status of the patient or the virulence of the pathogen. In the first group, *Pseudomonas aeruginosa* strains were isolated from five patients, and it was assumed that we were dealing with a situation where patients are affected by many severe co-morbidities and their immune system is impaired and undergoes complete collapse under the influence of infection. The second group of *Pseudomonas aeruginosa* strains were isolated from symptomatic patients with urinary tract infections, but without any comorbidity, and these patients were not immunocompromised. Their infections do not generalize into septicemia derived from the urinary tract, the so-called urosepsis. However, some kind of abnormal immune response may be suspected. The third group of patients were individuals who were asymptomatic carriers. In each of these groups, *Pseudomonas aeruginosa* infections with pathogens of different virulence may be expected, and analyses were performed to identify the main virulence factors of the bacterial strains in each of these groups. Conclusions on the association of immunological status of patients with virulence of individual strains of *Pseudomonas aeruginosa* must inevitably have to be indirect because we are unable to effectively, quickly and quantitatively determine the efficacy of the patient’s immune system as a whole—it is often difficult to pinpoint exactly where there is a deficiency of immunity in a patient without a congenital immune defect, which is associated with an opportunistic infection, and yet such deficiency is a fact. This difficulty also arises from the complexity of the immune system and the multiple interactions among its numerous components. We used PBMC of healthy donors to avoid the influence of the in vivo factors associated with patient condition, e.g., Systemic Inflammatory Response Syndrome in patients with systemic infections.

Phagocytosis of the *Pseudomonas aeruginosa* strains that colonize the urinary tract can have far-reaching consequences. This bacterium has a large predilection for adherence and growth in the form of biofilms on artificial surfaces in the body, including commonly used urinary catheters [10]. In addition, catheter insertion often causes mucosal damage to the mucous membranes with which it contacts, naturally promoting infection. Publications focused on infections derived from infected catheters are numerous, and the results show that about 40% of in-hospital infections are infections of the urinary tract associated with their presence [10]. Less work has been reported on the incidence of *Pseudomonas aeruginosa* in these cases, but it should be assumed that this is a fairly common bacterium that causes these infections. For example, in a retrospective Japanese study, Horino and colleagues reviewed 134 cases of *Pseudomonas aeruginosa* over seven years, where the most common illness in the infected people was leukemia, and the most common starting location was the urinary tract (24.6%) [11]. Ambulatory urinary tract infections with *Pseudomonas aeruginosa* are not uncommon, although this is definitely not the dominant bacterium in these cases. Sobczyk and colleagues estimate the incidence of urinary tract infections by this pathogen at 9% after *Escherichia coli* and *Staphylococcus saprophyticus* [12]. This information is related to outpatient infections in children. 

After the pathogen breaks through the non-specific immunity barrier, the infection can be countered by specific immunity, and it is this branch of the immune response that was assessed in our study. 

Dendritic cells are the most potent antigen presenting cells that are able to efficiently induce primary T-cell-mediated responses to pathogens, including bacteria. The induction of dendritic cell maturation was confirmed in previous research [13,14]. We found a statistically significant difference in percentage of mature DCs (CD83+CD1a-HLA-DR++) after incubation with *Pseudomonas aeruginosa* lysates between the strains isolated from the most severely ill patients and from asymptomatic carriers. Zhang et al. identified the *Pseudomonas aeruginosa* Mannose Sensitive Hemagglutination Strain as an important exogenous factor that induced DCs maturation toward a Th1-promoting phenotype [13]. Their results confirmed that some strains of *Pseudomonas aeruginosa* could be more efficient in the induction of DCs maturation. On the other hand, they generated dendritic cells from isolated CD14+ monocytes, and in our study, we used PBMC. In patients in poor general condition, where there is a collapse of the body’s defenses, it is highly probable that an ineffective response to this otherwise highly immunogenic *Pseudomonas aeruginosa* strain may be expected. Perhaps the differences observed are related to virulence of strains, so that bacteria that colonize the upper respiratory tract are potentially the least aggressive and are sufficiently controlled by the immune system also with minimal effort.

The increased expression of HLA-DR on the variety immune cells is associated with their activation and maturation. We found a significantly higher percentage of HLA-DR+ PBMC and highest expression after stimulation with the lysates of *Pseudomonas aeruginosa* isolated from critically ill patients. Avendaño-Ortiz J et al. showed that monocytes from patients with cystic fibrosis (CF) colonized by *Pseudomonas aeruginosa* exhibit a lower expression of HLA-DR+ (ratio between mean intensity of fluorescence (MIF) on LPS stimulated cells divided by MIF on unstimulated cells) than non-colonized patients [15]. The expression HLA-DR on monocytes of non-colonized patients was similar to the cells isolated from healthy volunteers. In present study, we also used cells isolated from healthy volunteers, and we can hypothesize that lower expression could be associated with massive and/or prolonged infections with *Pseudomonas aeruginosa*, rather than with a particular strain of this bacteria.

We did not find any statistically significant associations between features of examined *Pseudomonas aeruginosa* strains and percentages of CD3+CD4+ cells after stimulation with bacterial lysates. The median percentages of CD3+CD4+ cells ranged from 34.37% to 42.78%. The lower percentage of CD3+CD4+ after incubation with lysates of particular strains could be associated with a higher concentration of alkaline protease (AP) or elastase (Ela) in the lysates. Pedersen et al. found that AP and Ela could specifically cleave CD4 molecules [16].

There are works in the literature on the role of IL-17 in the immune response to infections caused by *Pseudomonas aeruginosa*. Liu and co-workers demonstrated a significant role for IL-17 in murine models, in addition to IL-22 and IL-23. In the development of acute inflammation of the lungs [17], the main sources of this cytokine were T γδ lymphocytes. Similar results were obtained by Chinese scientists Xu and colleagues [18]; based on the conducted experiments, they concluded that IL-17 and cytokines induced by it play a role in the etiology of *Pseudomonas aeruginosa* pneumonia. IL-17 plays a protective role in the early stages of infection, and its concentration rapidly increases during infection [19]. The high levels of this cytokine, by strong induction of inflammation, can indirectly adversely affect the damaged tissue of an organism by excessive or persistent inflammation. In our study, it was found that all bacterial lysates stimulated Th17 lymphocytes, but no significant differences in stimulation potentials between *Pseudomonas aeruginosa* strains were found. Evaluation of the specific immune response to the tested strains of *Pseudomonas aeruginosa* brought several important conclusions. The first is that the specific response is less dependent on the features of particular *Pseudomonas aeruginosa* strains isolated from patients with different clinical courses of infection. This demonstrates that the specific response expressed in the effector phase is rather not significantly impaired by any of the examined strains of *Pseudomonas aeruginosa*. More detailed research is still underway on the extent to which the resulting antibody neutralizes the pathogen (as it may change its phenotype during infection) and to evaluate the response of the specific immune system to live bacteria. Creating a laboratory model of sepsis or bacteremia *Pseudomonas aeruginosa* remains an interesting experimental challenge.

In most patients, especially those infected by the pathogen, it is difficult to suggest an immune system test evaluating specific immune responses that would clearly indicate an immune system defect. In spite of these difficulties, it seems that we should not be satisfied with the diagnosis of the infection itself, but we should look for general and local factors that predispose the patient to it, especially if the patient is in a fairly good general condition or is suffering from recurrent infections. It may be possible to deepen our knowledge about the still unclear etiology of opportunistic infections and to identify factors favoring such infections both on the host side and on the pathogen side.

## 5. Conclusions

The most important factor in favor of severe generalized infections caused by *Pseudomonas aeruginosa* is probably the poor general condition of the patient. The induction of specific immune response to the tested strains of *Pseudomonas aeruginosa* is maintained when healthy donor cells are used. This provides a preliminary basis for considering the use of vaccination in patients with recurrent infections and coexisting risk factors. Moreover, strains responsible for the most severe infections effectively stimulate the immune system. In patients with good general condition but with recurrent infections of *Pseudomonas aeruginosa*, it seems appropriate to seek general and local factors conducive to the onset of this infection. Additional information may be provided by studies of immunity specific to live bacteria in laboratory models of infection caused by *Pseudomonas aeruginosa*.

## Figures and Tables

**Table 1:** Analysis of virulence genes profiles and grouping of isolated *Pseudomonas aeruginosa* strains.

	*algD*	*lasB*	*toxA*	*plcH*	*plcN*	*exoS*	*nan1*	Group
Pa 1	+	+	+	+	+	−	+	1
Pa 2	+	+	+	+	+	+	+	2
Pa 3	+	+	+	+	+	+	+	2
Pa 4	+	+	+	+	+	−	−	3
Pa 5	+	+	+	+	+	+	+	2
Pa 6	+	+	+	+	+	+	+	2
Pa 7	+	+	+	+	+	+	+	2
Pa 8	+	+	+	+	+	+	+	2
Pa 9	+	+	+	+	+	−	−	3
Pa 10	+	+	+	+	+	+	−	4
Pa 11	+	+	+	+	+	−	+	1
Pa 12	+	+	+	+	+	+	-	4
Pa 13	+	+	+	+	+	−	−	3
Pa 14	+	+	+	+	+	+	+	2
Pa 15	+	+	+	+	+	+	+	2

## Data Availability

The data presented in this study are available upon request from the corresponding author. The data are not publicly available, as they include sensitive clinical data.

## Supplementary Materials

The following are available online at https://www.mdpi.com/article/10.3390/cells11010003/s1. Figure S1: The representative example of flow cytometric analysis of HLA-DR expression and dendritic cells maturation. Figure S2: The representative example of flow cytometric analysis of percentage of Th17 lymphocytes. Table S1: The sequences of primers used in assessment of virulence genes expression. Table S2: Difference in percentage (%) of mature (CD83+CD1a-HLA-DR++) dendritic cells after stimulation with bacterial lysates between individual patients. Table S3: Difference in percentage (%) of partially matured (CD83+CD1a+HLA-DR+) dendritic cells after stimulation with bacterial lysates between individual patients. Table S4: Difference in percentage (%) of immature (CD83-CD1a+ HLA–DR+/low) dendritic cells after stimulation with bacterial lysates between individual patients. Table S5: Difference in percentage (%) of HLA-DR+ PBMCs in culture-stimulated lysates. Table S6: Difference in expression of HLA-DR on PBMCs-MFI. Table S7: Differences in percentages of CD3+CD4+ cells. Table S8: Differences in percentages of CD3+CD4+IL-17+ cells.
