# Supplementary material for: Evaluation of Selected Parameters of the Specific Immune Response against Pseudomonas aeruginosa Strains
Source: Cells. 2021 Dec 21;11(1):3. doi: 10.3390/cells11010003 (PMC8750466; doi:10.3390/cells11010003)
Supplement: Supplementary file 1 [file cells-11-00003-s001.zip › Supplementary Figure S1.pdf]

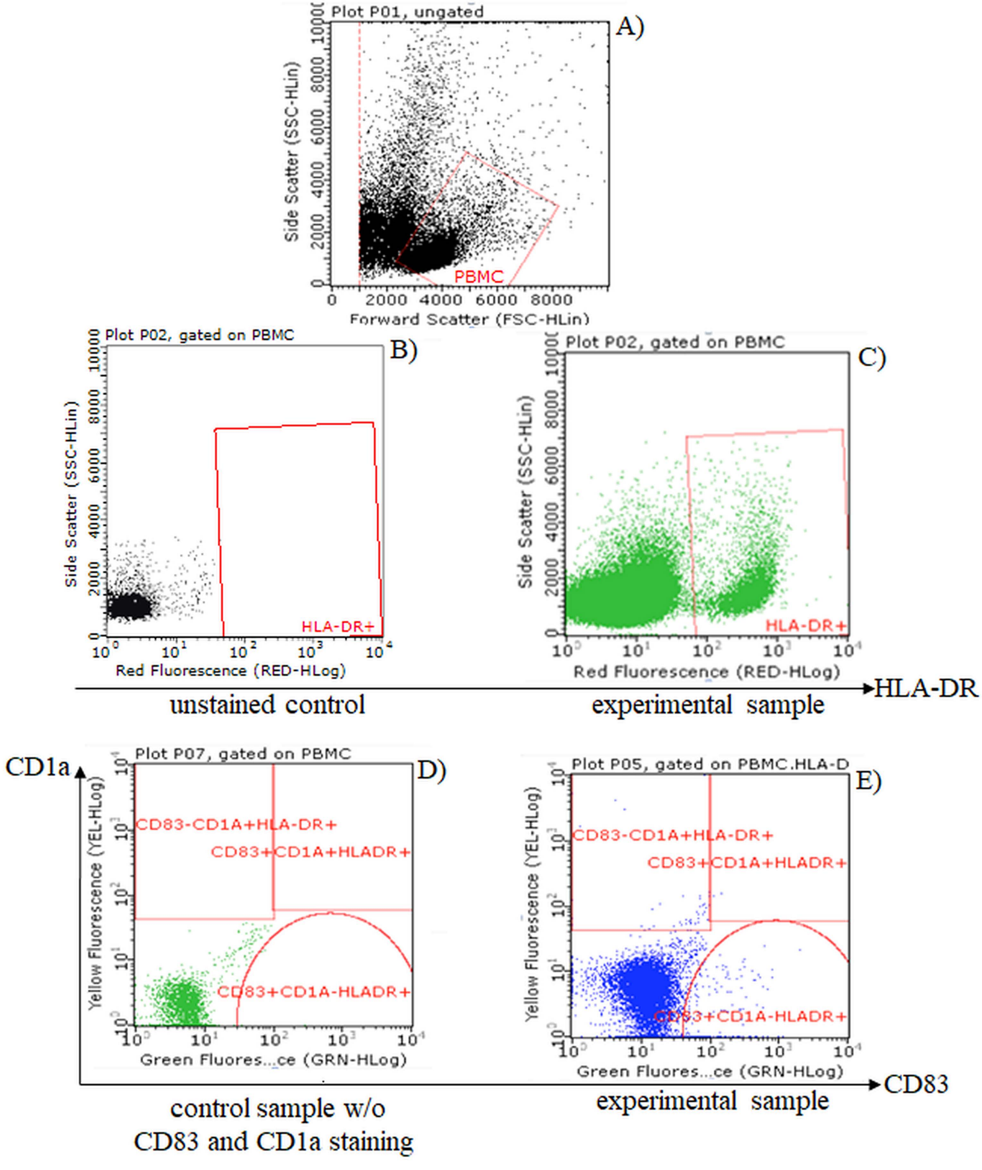

Plot P01, ungated
A)
Side Scatter (SSC-HLin)
Forward Scatter (FSC-HLin)
PBMC
Plot P02, gated on PBMC
B)
Side Scatter (SSC-HLin)
Red Fluorescence (RED-HLog)
HLA-DR+
Plot P02, gated on PBMC
C)
Side Scatter (SSC-HLin)
Red Fluorescence (RED-HLog)
HLA-DR+
HLA-DR
unstained control
experimental sample
CD1a
Plot P07, gated on PBMC
D)
Yellow Fluorescence (YEL-HLog)
CD83-CD1A+HLA-DR+
CD83+CD1A+HLADR+
CD83+CD1A-HLADR+
Green Fluores...ce (GRN-HLog)
Plot P05, gated on PBMC.HLA-D
E)
Yellow Fluorescence (YEL-HLog)
CD83-CD1A+HLA-DR+
CD83+CD1A+HLADR+
CD83+CD1A-HLADR+
Green Fluores...ce (GRN-HLog)
CD83
control sample w/o
CD83 and CD1a staining
experimental sample
