# Supplementary material for: Evaluation of Selected Parameters of the Specific Immune Response against Pseudomonas aeruginosa Strains
Source: Cells. 2021 Dec 21;11(1):3. doi: 10.3390/cells11010003 (PMC8750466; doi:10.3390/cells11010003)
Supplement: Supplementary file 1 [file cells-11-00003-s001.zip › Supplementary Table S1.pdf]

Table S1: The sequences of primers used in assessment of virulence genes expression.

Gene	primers	Nucleotide sequence	Amplicon size [bp]
algD	forward	ATGCGAATCAGCATCTTTGGT	1310
	reverse	CTACCAGCAGATGCCCTCGGC	
lasB	forward	GGAATGAACGAAGCGTTCTC	300
	reverse	GGTCCAGTAGTAGCGGTTGG	
toxA	forward	GGTAACCAGCTCAGCCACAT	352
	reverse	TGATGTCCAGGTCATGCTTC	
plcH	forward	GGAAGCCATGGGCTACTTCAA	307
	reverse	AGAGTGACGAGGAGCGGTAG	
plcN	forward	GTTATCGCAACCAGCCCTAC	466
	reverse	AGGTCGAACACCTGGAACAC	
exoS	forward	CTTGAAGGGACTCGACAAGG	504
	reverse	TTCAGGTCCGCGTAGTGAAT	
nan1	forward	AGGATGAATACTTATTTTGAT	1316
	reverse	TCACTAAATCCATCTCTGACCCGATA	
