# Supplementary material for: Evaluation of Selected Parameters of the Specific Immune Response against Pseudomonas aeruginosa Strains
Source: Cells. 2021 Dec 21;11(1):3. doi: 10.3390/cells11010003 (PMC8750466; doi:10.3390/cells11010003)
Supplement: Supplementary file 1 [file cells-11-00003-s001.zip › Supplementary Table S2.pdf]

Table S1: Difference in percentage [%] of mature (CD83+CD1a-HLA–DR++) dendritic cells after stimulation with bacterial lysates between individual patients.

Difference in percentage [%] of mature dendritic cells in lysate-stimulated cultures. χ^2 ANOVA = 33.75 p<0.00224															
	Pa 1	Pa 2	Pa 3	Pa 4	Pa 5	Pa 6	Pa 7	Pa 8	Pa 9	Pa 10	Pa 11	Pa 12	Pa 13	Pa 14	Pa 15
Pa 1	-	NS	NS	NS	NS	NS	NS	NS	NS	NS	NS	NS	NS	NS	NS
Pa 2	NS	-	NS	NS	NS	NS	NS	NS	NS	NS	NS	NS	NS	NS	NS
Pa 3	NS	NS	-	NS	NS	NS	NS	NS	NS	NS	NS	NS	NS	NS	NS
Pa 4	NS	NS	NS	-	NS	NS	NS	NS	NS	NS	NS	NS	NS	NS	NS
Pa 5	NS	NS	NS	NS	-	NS	NS	NS	NS	NS	NS	NS	NS	NS	NS
Pa 6	NS	NS	NS	NS	NS	-	NS	NS	NS	NS	NS	NS	NS	NS	NS
Pa 7	NS	NS	NS	NS	NS	NS	-	NS	NS	NS	NS	NS	NS	NS	NS
Pa 8	NS	NS	NS	NS	NS	NS	NS	-	NS	NS	NS	NS	NS	NS	NS
Pa 9	NS	NS	NS	NS	NS	NS	NS	NS	-	NS	NS	NS	NS	NS	NS
Pa 10	NS	NS	NS	NS	NS	NS	NS	NS	NS	-	NS	NS	NS	NS	NS
Pa 11	NS	NS	NS	NS	NS	NS	NS	NS	NS	NS	-	NS	NS	NS	NS
Pa 12	NS	NS	NS	NS	NS	NS	NS	NS	NS	NS	NS	-	NS	NS	NS
Pa 13	NS	NS	NS	NS	NS	NS	NS	NS	NS	NS	NS	NS	-	NS	NS
Pa 14	NS	NS	NS	NS	NS	NS	NS	NS	NS	NS	NS	NS	NS	-	NS
Pa 15	NS	NS	NS	NS	NS	NS	NS	NS	NS	NS	NS	NS	NS	NS	-
No.	Pa 1	Pa 2	Pa 3	Pa 4	Pa 5	Pa 6	Pa 7	Pa 8	Pa 9	Pa 10	Pa 11	Pa 12	Pa 13	Pa 14	Pa 15
median	16.5	15.78	12.97	13.56	10.84	12.79	11.33	10.06	10.31	10.26	9.27	9.23	10.48	10.0	8.83
IQR	12,56	3,34	8.76	2.86	5.71	6.19	6.3	3.9	3.96	5.71	4.09	11.23	6.5	7.06	6.16
