# Supplementary material for: Evaluation of Selected Parameters of the Specific Immune Response against Pseudomonas aeruginosa Strains
Source: Cells. 2021 Dec 21;11(1):3. doi: 10.3390/cells11010003 (PMC8750466; doi:10.3390/cells11010003)
Supplement: Supplementary file 1 [file cells-11-00003-s001.zip › Supplementary Table S3.pdf]

Table S2: Difference in percentage [%] of partially matured (CD83+CD1a+HLA–DR+) dendritic cells after stimulation with bacterial lysates between individual patients.

Difference in percentage [%] of partially matured dendritic cells in lysate-stimulated cultures.

χ2 ANOVA = 18.53 p<0.18379

	Pa 1	Pa 2	Pa 3	Pa 4	Pa 5	Pa 6	Pa 7	Pa 8	Pa 9	Pa 10	Pa 11	Pa 12	Pa 13	Pa 14	Pa 15
Pa 1	-	NS	NS	NS	NS	NS	NS	NS	NS	NS	NS	NS	NS	NS	NS
Pa 2	NS	-	NS	NS	NS	NS	NS	NS	NS	NS	NS	NS	NS	NS	NS
Pa 3	NS	NS	-	NS	NS	NS	NS	NS	NS	NS	NS	NS	NS	NS	NS
Pa 4	NS	NS	NS	-	NS	NS	NS	NS	NS	NS	NS	NS	NS	NS	NS
Pa 5	NS	NS	NS	NS	-	NS	NS	NS	NS	NS	NS	NS	NS	NS	NS
Pa 6	NS	NS	NS	NS	NS	-	NS	NS	NS	NS	NS	NS	NS	NS	NS
Pa 7	NS	NS	NS	NS	NS	NS	-	NS	NS	NS	NS	NS	NS	NS	NS
Pa 8	NS	NS	NS	NS	NS	NS	NS	-	NS	NS	NS	NS	NS	NS	NS
Pa 9	NS	NS	NS	NS	NS	NS	NS	NS	-	NS	NS	NS	NS	NS	NS
Pa 10	NS	NS	NS	NS	NS	NS	NS	NS	NS	-	NS	NS	NS	NS	NS
Pa 11	NS	NS	NS	NS	NS	NS	NS	NS	NS	NS	-	NS	NS	NS	NS
Pa 12	NS	NS	NS	NS	NS	NS	NS	NS	NS	NS	NS	-	NS	NS	NS
Pa 13	NS	NS	NS	NS	NS	NS	NS	NS	NS	NS	NS	NS	-	NS	NS
Pa 14	NS	NS	NS	NS	NS	NS	NS	NS	NS	NS	NS	NS	NS	-	NS
Pa 15	NS	NS	NS	NS	NS	NS	NS	NS	NS	NS	NS	NS	NS	NS	-
No.	Pa 1	Pa 2	Pa 3	Pa 4	Pa 5	Pa 6	Pa 7	Pa 8	Pa 9	Pa 10	Pa 11	Pa 12	Pa 13	Pa 14	Pa 15
median	0.00	0.21	0.00	0.00	0.1	0.09	0.00	0.07	0.00	0.00	0.00	0.00	0.00	0.1	0.00
IQR	0.06	0.46	0.00	0.13	0.17	0.15	0.13	0.28	0.1	0.06	0.23	0.14	0.00	0.2	0.00
