# Supplementary material for: Evaluation of Selected Parameters of the Specific Immune Response against Pseudomonas aeruginosa Strains
Source: Cells. 2021 Dec 21;11(1):3. doi: 10.3390/cells11010003 (PMC8750466; doi:10.3390/cells11010003)
Supplement: Supplementary file 1 [file cells-11-00003-s001.zip › Supplementary Table S4.pdf]

Table S3: Difference in percentage [%] of immature (CD83-CD1a+ HLA–DR+/low) dendritic cells after stimulation with bacterial lysates between individual patients.

Difference in percentage [%] of partially immature dendritic cells in lysate-stimulated cultures.															
**χ2ANOVA = 16.23 p<0.29919**															
	Pa 1	Pa 2	Pa 3	Pa 4	Pa 5	Pa 6	Pa 7	Pa 8	Pa 9	Pa 10	Pa 11	Pa 12	Pa 13	Pa 14	Pa 15
Pa 1	-	NS	NS	NS	NS	NS	NS	NS	NS	NS	NS	NS	NS	NS	NS
Pa 2	NS	-	NS	NS	NS	NS	NS	NS	NS	NS	NS	NS	NS	NS	NS
Pa 3	NS	NS	-	NS	NS	NS	NS	NS	NS	NS	NS	NS	NS	NS	NS
Pa 4	NS	NS	NS	-	NS	NS	NS	NS	NS	NS	NS	NS	NS	NS	NS
Pa 5	NS	NS	NS	NS	-	NS	NS	NS	NS	NS	NS	NS	NS	NS	NS
Pa 6	NS	NS	NS	NS	NS	-	NS	NS	NS	NS	NS	NS	NS	NS	NS
Pa 7	NS	NS	NS	NS	NS	NS	-	NS	NS	NS	NS	NS	NS	NS	NS
Pa 8	NS	NS	NS	NS	NS	NS	NS	-	NS	NS	NS	NS	NS	NS	NS
Pa 9	NS	NS	NS	NS	NS	NS	NS	NS	-	NS	NS	NS	NS	NS	NS
Pa 10	NS	NS	NS	NS	NS	NS	NS	NS	NS	-	NS	NS	NS	NS	NS
Pa 11	NS	NS	NS	NS	NS	NS	NS	NS	NS	NS	-	NS	NS	NS	NS
Pa 12	NS	NS	NS	NS	NS	NS	NS	NS	NS	NS	NS	-	NS	NS	NS
Pa 13	NS	NS	NS	NS	NS	NS	NS	NS	NS	NS	NS	NS	-	NS	NS
Pa 14	NS	NS	NS	NS	NS	NS	NS	NS	NS	NS	NS	NS	NS	-	NS
Pa 15	NS	NS	NS	NS	NS	NS	NS	NS	NS	NS	NS	NS	NS	NS	-
No.	Pa 1	Pa 2	Pa 3	Pa 4	Pa 5	Pa 6	Pa 7	Pa 8	Pa 9	Pa 10	Pa 11	Pa 12	Pa 13	Pa 14	Pa 15
median	0.15	0.00	0.05	0.00	0.00	0.15	0.11	0.00	0.00	0.00	0.00	0.00	0.00	0.05	0.00
IQR	0.23	0.27	0.23	0.14	0.39	0.32	0.29	0.14	0.43	0.11	0.00	0.32	0.24	0.41	0.16
