# Supplementary material for: Evaluation of Selected Parameters of the Specific Immune Response against Pseudomonas aeruginosa Strains
Source: Cells. 2021 Dec 21;11(1):3. doi: 10.3390/cells11010003 (PMC8750466; doi:10.3390/cells11010003)
Supplement: Supplementary file 1 [file cells-11-00003-s001.zip › Supplementary Table S5.pdf]

Table S4: Difference in percentage [%] of HLA-DR+ PBMCs in culture-stimulated lysates.

**Difference in percentage [%] of HLA-DR+ PBMCs in culture-stimulated lysates** **$\chi 2$ ANOVA = 19.06 p<0.16272**															
	Pa 1	Pa 2	Pa 3	Pa 4	Pa 5	Pa 6	Pa 7	Pa 8	Pa 9	Pa 10	Pa 11	Pa 12	Pa 13	Pa 14	Pa 15
Pa 1	-	NS	NS	NS	NS	NS	NS	NS	NS	NS	NS	NS	NS	NS	NS
Pa 2	NS	-	NS	NS	NS	NS	NS	NS	NS	NS	NS	NS	NS	NS	NS
Pa 3	NS	NS	-	NS	NS	NS	NS	NS	NS	NS	NS	NS	NS	NS	NS
Pa 4	NS	NS	NS	-	NS	NS	NS	NS	NS	NS	NS	NS	NS	NS	NS
Pa 5	NS	NS	NS	NS	-	NS	NS	NS	NS	NS	NS	NS	NS	NS	NS
Pa 6	NS	NS	NS	NS	NS	-	NS	NS	NS	NS	NS	NS	NS	NS	NS
Pa 7	NS	NS	NS	NS	NS	NS	-	NS	NS	NS	NS	NS	NS	NS	NS
Pa 8	NS	NS	NS	NS	NS	NS	NS	-	NS	NS	NS	NS	NS	NS	NS
Pa 9	NS	NS	NS	NS	NS	NS	NS	NS	-	NS	NS	NS	NS	NS	NS
Pa 10	NS	NS	NS	NS	NS	NS	NS	NS	NS	-	NS	NS	NS	NS	NS
Pa 11	NS	NS	NS	NS	NS	NS	NS	NS	NS	NS	-	NS	NS	NS	NS
Pa 12	NS	NS	NS	NS	NS	NS	NS	NS	NS	NS	NS	-	NS	NS	NS
Pa 13	NS	NS	NS	NS	NS	NS	NS	NS	NS	NS	NS	NS	-	NS	NS
Pa 14	NS	NS	NS	NS	NS	NS	NS	NS	NS	NS	NS	NS	NS	-	NS
Pa 15	NS	NS	NS	NS	NS	NS	NS	NS	NS	NS	NS	NS	NS	NS	-
No.	Pa 1	Pa 2	Pa 3	Pa 4	Pa 5	Pa 6	Pa 7	Pa 8	Pa 9	Pa 10	Pa 11	Pa 12	Pa 13	Pa 14	Pa 15
median	3.52	5.47	4.76	4.21	4.34	4.35	4.33	4.66	4.54	4.31	3.86	3.83	4.11	3.46	3.97
IQR	2.54	2.66	2.9	1.13	1.04	1.17	1.2	2.66	1.96	1.66	1.82	2.53	2.44	2.3	0.72
