# Supplementary material for: Evaluation of Selected Parameters of the Specific Immune Response against Pseudomonas aeruginosa Strains
Source: Cells. 2021 Dec 21;11(1):3. doi: 10.3390/cells11010003 (PMC8750466; doi:10.3390/cells11010003)
Supplement: Supplementary file 1 [file cells-11-00003-s001.zip › Supplementary Table S7.pdf]

Table S6: Differences in percentages of CD3+CD4+ cells.

Differences in percentages of CD3+CD4+ cells [%] $\chi 2$ ANOVA = 40.266 p<0.00023															
	Pa 1	Pa 2	Pa 3	Pa 4	Pa 5	Pa 6	Pa 7	Pa 8	Pa 9	Pa 10	Pa 11	Pa 12	Pa 13	Pa 14	Pa 15
Pa 1	-	NS	NS	NS	NS	NS	NS	p<0.05	NS	NS	NS	NS	NS	NS	p<0.05
Pa 2	NS	-	NS	NS	NS	NS	NS	NS	NS	NS	NS	NS	NS	NS	NS
Pa 3	NS	NS	-	NS	NS	NS	NS	NS	NS	NS	NS	NS	NS	NS	NS
Pa 4	NS	NS	NS	-	NS	NS	NS	NS	NS	NS	NS	NS	NS	NS	NS
Pa 5	NS	NS	NS	NS	-	NS	NS	NS	NS	NS	NS	NS	NS	NS	NS
Pa 6	NS	NS	NS	NS	NS	-	NS	NS	NS	NS	NS	NS	NS	NS	NS
Pa 7	NS	NS	NS	NS	NS	NS	-	NS	NS	NS	NS	NS	NS	NS	NS
Pa 8	p<0.05	NS	NS	NS	NS	NS	NS	-	NS	NS	NS	NS	NS	NS	NS
Pa 9	NS	NS	NS	NS	NS	NS	NS	NS	-	NS	NS	NS	NS	NS	NS
Pa 10	NS	NS	NS	NS	NS	NS	NS	NS	NS	-	NS	NS	NS	NS	NS
Pa 11	NS	NS	NS	NS	NS	NS	NS	NS	NS	NS	-	NS	NS	NS	NS
Pa 12	NS	NS	NS	NS	NS	NS	NS	NS	NS	NS	NS	-	NS	NS	NS
Pa 13	NS	NS	NS	NS	NS	NS	NS	NS	NS	NS	NS	NS	-	NS	NS
Pa 14	NS	NS	NS	NS	NS	NS	NS	NS	NS	NS	NS	NS	NS	-	NS
Pa 15	p<0.05	NS	NS	NS	NS	NS	NS	NS	NS	NS	NS	NS	NS	NS	-
No.	Pa 1	Pa 2	Pa 3	Pa 4	Pa 5	Pa 6	Pa 7	Pa 8	Pa 9	Pa 10	Pa 11	Pa 12	Pa 13	Pa 14	Pa 15
median	41.41	42.67	34.37	41.55	41.42	42.78	42.78	40.51	41.92	39.72	42.27	42.36	41.07	40.03	40.53
IQR	13.81	12.45	14.47	13.84	14.7	12.24	13.03	13.11	18.11	16.91	14.11	11.56	14.45	13.79	18.2
