# Supplementary figures and images for: Evaluation of Selected Parameters of the Specific Immune Response against Pseudomonas aeruginosa Strains
Source: Cells. 2021 Dec 21;11(1):3. doi: 10.3390/cells11010003 (PMC8750466; doi:10.3390/cells11010003)

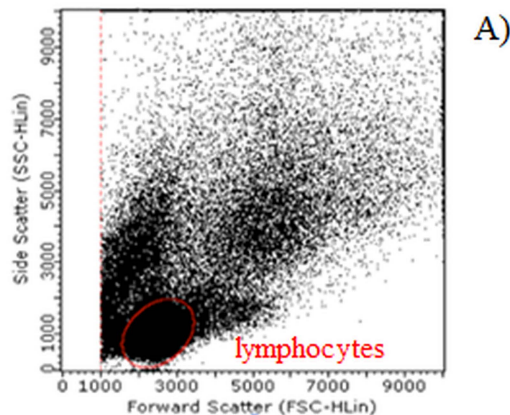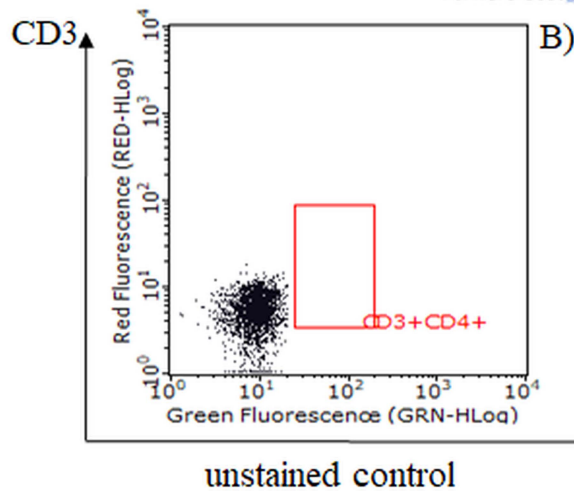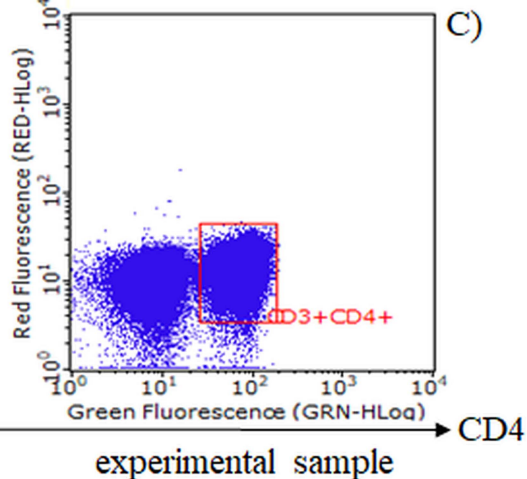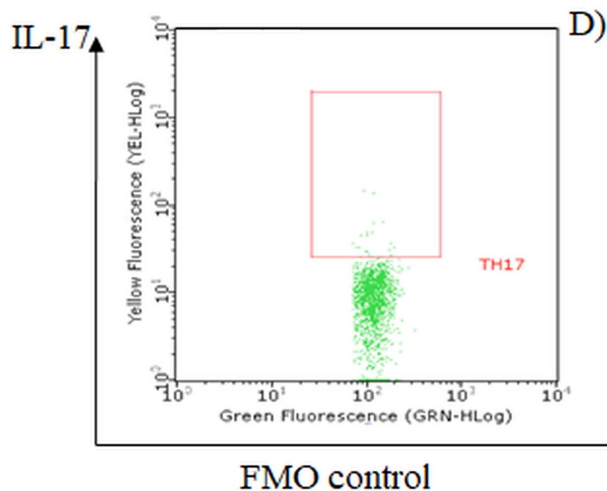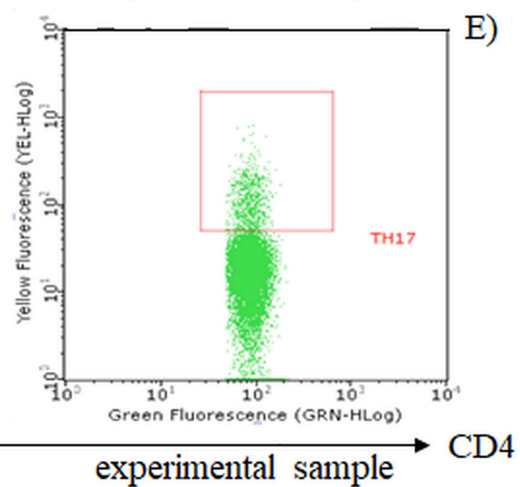

Supplement: Supplementary file 1 [file cells-11-00003-s001.zip › Supplementary Figure S2.pdf]
